# Liver‐tumor mimics as a potential translational framework for planning and testing irreversible electroporation with multiple electrodes

**DOI:** 10.1002/btm2.10607

**Published:** 2023-11-23

**Authors:** Adriana Leticia Vera‐Tizatl, Regine van der Hee, Jeroen Cornelissen, Claudia Elizabeth Vera‐Tizatl, Momen Abayazid, Jurgen J. Fütterer

**Affiliations:** ^1^ Department of Electrical Engineering, Mathematics and Computer Sciences University of Twente Enschede The Netherlands; ^2^ Department of Medical Imaging, Faculty of Sciences and Technology, Biomolecular NanoTechnology Group University of Twente Enschede The Netherlands; ^3^ Department of Infectomics and Molecular Pathogenesis Center for Research and Advanced Studies of the National Polytechnic Institute Mexico City Mexico; ^4^ Department of Medical Imaging Radboudumc Nijmegen The Netherlands

**Keywords:** co‐cultured hydrogels, computational treatment planning, hepatocellular carcinoma, Hep‐G2, HUVEC, hydrogel tumor

## Abstract

Irreversible electroporation (IRE) has emerged as an appealing non‐ionizing, non‐thermal ablation therapy, independent of antineoplastic drugs. Limited but successful outcomes in IRE conducted in vivo, in small focal hepatocellular carcinomas (HCC), have been reported. Nonetheless, the electric parameters of IRE are usually delivered in an unplanned manner. This work investigates the integration of computational modeling to hydrogels mimicking the HCC microenvironment, as a powerful framework to: circumvent ethical concerns of in vivo experimentation; safely tune the electric parameters reaching the IRE electric field threshold; and propel the translation of IRE as a routine clinical alternative to the treatment of HCC. Therefore, a parametric study served to evaluate the effects of the pulse amplitude, the number of pulses and electrodes, the treatment time, the hydrogel–tumor size, and the cell type. The ablation extent was surveyed by confocal microscopy and magnetic resonance imaging (MRI) in cylindrical and realistic tumor‐shaped hydrogels, respectively. A large ablation (70%–100%) was verified in all constructs.

**Translational Impact Statement**

This investigation describes the computational planning, delivery, and effectiveness of IRE in hydrogels recapitulating key elements of the HCC microenvironment. It builds on exhibiting the correlation between theoretical and experimental ablation induced by pre‐planned IRE. The in vitro setup herein circumvents the ethical difficulties related to in vivo experimentation and targets the translation of IRE as a routine clinical alternative to the treatment of HCC.

## 1 INTRODUCTION

Worldwide, liver cancer is a concerning pathology, frequently with poor prognoses due to its diagnosis in advanced stages.^1^, ^2^ Hepatocellular carcinoma (HCC) is the most persistent type, representing more than 90% of the primary liver cancers, for which systemic toxic chemotherapy with immunotherapy are mainly offered to the patients to counteract the multidrug resistance.^2^ Yet for early diagnoses, partial liver resection is suitable for only 5%–10% of patients with a small (<2 cm) nonmetastatic disease from which half of them recur within 2 years after surgery.^3^ Next to liver surgery, numerous ablation therapies have come to light as curative alternatives and as a means of sustainability of hepatic tissue, for example, radiofrequency ablation (RFA), microwave ablation (MWA), cryoablation, localized drug delivery with electrochemotherapy (ECT), and irreversible electroporation (IRE).^1^, ^4^, ^5^ At present, RFA is the most used ablation therapy in HCC treatment, showing a local recurrence up to 31%. Nonetheless, IRE is being increasingly preferred since it has reduced the local recurrence to 7.5% while lacking the heat‐sink effect.^4^ Moreover, IRE exhibits advantages over other ablative methods for soft tissue since it is a non‐ionizing non‐thermal localized process, which is most suitable in neoplasia located near critical structures such as blood vessels, major bile ducts, and nerves, which must be preserved for further angiogenesis.^1^, ^4^, ^6^, ^7^, ^8^

IRE induces the permanent permeabilization of the cell membrane, provoking a necrotic process after its exposure to a sufficiently high pulsed electric field (EF).^9^, ^10^, ^11^ IRE consists of the local delivery of high‐voltage electric pulses through needle‐electrodes placed in the target neoplasia. The planning of the therapy by the numerical calculation of the ablation volume based on the EF threshold for IRE of the tissue of interest is actively investigated.^10^, ^12^ The efficacy of IRE in humans has been reported in two main studies treating single small (< 25 mm) focal HCC.^7^, ^13^ These studies reported a no recurrence period of 10 months^7^ and 4 months, in addition to a 6‐month progression‐free survival of 74%.^13^ However, no information about the planning of the electric protocol, that is, pulse amplitude, number of pulses, pulse delivery frequency, and number of electrodes, was described.

**FIGURE 1:**
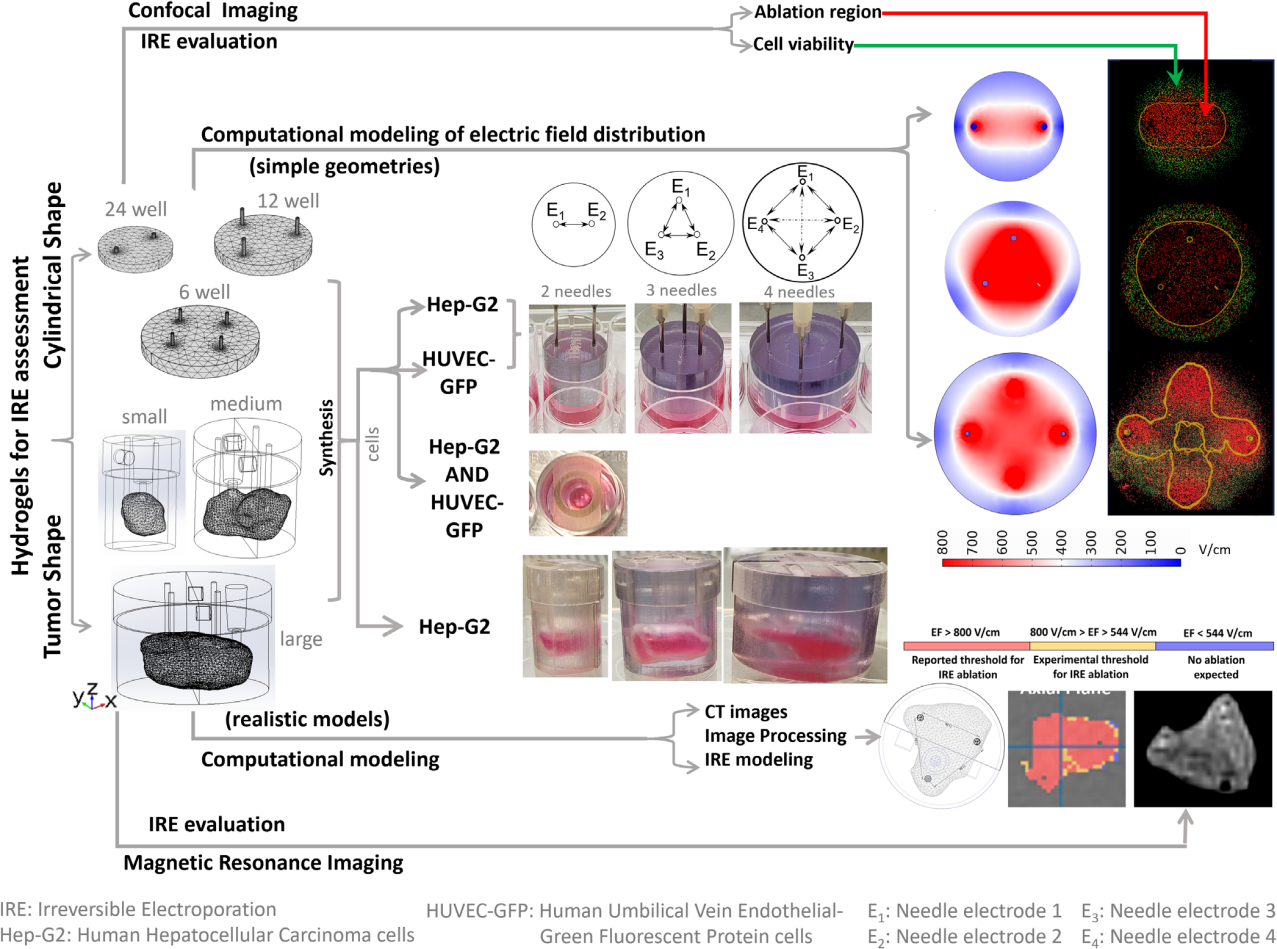
Workflow for the synthesis of hydrogels mimicking the HCC microenvironment and the evaluation of IRE by confocal microscopy and magnetic resonance imaging (MRI).

In this regard, this investigation aims at determining whether hydrogels may be utilized as reproducible in vitro models exhibiting the HCC phenotype to examine the ablation caused by computationally‐tuned IRE with two, three, and four needle‐electrodes, as depicted in Figure 1. The constructs presented hereby recapitulate three main features in the HCC microenvironment, that is, Hep‐G2 cells that are isolated from a human liver HCC; Matrigel, as the extracellular matrix, mimicking the basement membrane and maintaining differentiation states of cancer cells; and human umbilical vein endothelial cells (HUVEC) as a representative model of the human vascular endothelium.^14^, ^15^, ^16^ Figure 1 exhibits the synthesis of hydrogels in two ways to cope with the visualization of the cell‐death extent after IRE, that is, as cylindrical scaffolds cultured in well‐plates, being scanned by confocal microscopy; and as tumor‐shaped hydrogels, being imaged by magnetic resonance (MR).

## 2 RESULTS

### 2.1 Global evaluation of IRE in cylindrical hydrogels

Trial electric protocols were selected from computational modeling of the EF distribution in 24‐well, 190 mm^2^ hydrogels, treated with two needles. Pulse amplitude ranging from low (600 V) to high voltage (2300 V), estimated small to large ablation regions, respectively.

**FIGURE 2:**
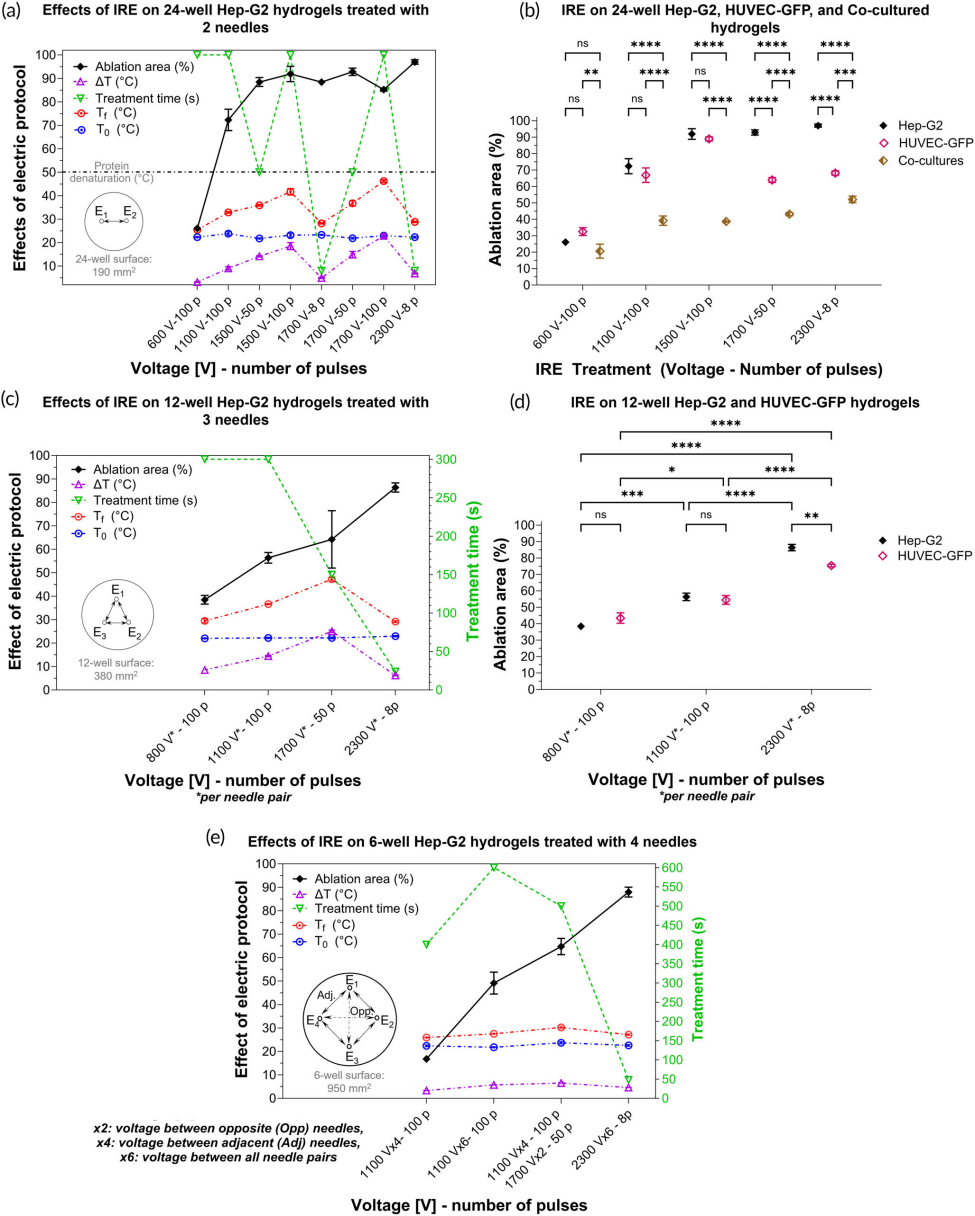
Effects of IRE on cylindrical hydrogels. (a) Impact of trial electric protocols on the ablation and temperature increase in 24‐well Hep‐G2 scaffolds when varying pulse amplitude and number of pulses. Statistically representative treatments were delivered for comparative analysis to: (b) 24‐well HUVEC‐GFP, and co‐cultured hydrogels; 12‐well (c) Hep‐G2 and (d) HUVEC‐GFP hydrogels treated with three needles; and (e) six‐well Hep‐G2 hydrogels treated with four needles. The treatment time is shown as an additional factor to determine preference of a treatment; 100‐μs pulse‐width and 1 Hz pulse‐delivery frequency remained constant parameters for all treatments.

Experimentally, 100‐μs pulse‐width and 1 Hz pulse‐delivery frequency were constant parameters in all treatments. Conversely, a parametric study varying the number of pulses was conducted for 1500 V and 1700 V, producing eight trial treatments in total, tested in 24‐well Hep‐G2 scaffolds (Figure 2a). The resulting ablation, temperature increase (ΔT) in the center of the hydrogel, and the duration of the treatment is plotted in Figure 2a. The effective number of pulses for 1500 and 1700 V was statistically determined based on the largest ablation region, lowest ΔT, and shortest treatment time. Therefore, five out of the eight trial protocols were delivered for comparative analysis to 24‐well HUVEC‐green fluorescent protein (GFP) and co‐cultured (Hep‐G2 + HUVEC‐GFP cells) hydrogels, as shown in Figure 2b. Therein, a significant ablation reduction was observed in endothelial hydrogels, and in co‐cultured hydrogels mostly. No statistical difference in the ablation extent was calculated between 1500 V‐100 pulses and 1700 V‐50 pulses. However, the treatment time and ΔT are significantly reduced with 1700 V‐50 pulses.

Hence, 1500 V‐100 pulses were discarded in cylindrical, 12‐well, 380 mm^2^, Hep‐G2 constructs, as shown in Figure 2c. Similarly, 800 V‐100 pulses were delivered instead of 600 V‐100 pulses, since this latter protocol computationally suggested a negligible ablation in 12‐well hydrogels, treated with three needles. Only three IRE protocols, representative of small, moderate, and large ablation, were delivered to 12‐well HUVEC‐GFP hydrogels, as shown in Figure 2d. Therein, a significant ablation reduction was observed in endothelial hydrogels for a high voltage.

The treatment of six‐well, 950 mm^2^ scaffolds with four needles was based on the number of possible active needle pairs and the distance between the electrodes, as depicted in Figure 2e. By activating adjacent (Adj) needles (1.4 cm electrode interspace) only, four electrode‐pairs may be activated. Activating the remaining opposite (Opp) needles (2 cm electrode interspace) results in six electrode‐pairs.

Due to the quiescent behavior of HUVEC‐GFP cells,^14^ a slow growth rate and low cell density were observed in HUVEC‐GFP cell culturing. Consequently, the co‐cultured framework was tested in 24‐well hydrogels, and experiments in six‐well hydrogels were conducted in Hep‐G2 constructs only.

### 2.2 Effects of IRE on 24‐well hydrogels treated with two needles

**FIGURE 3:**
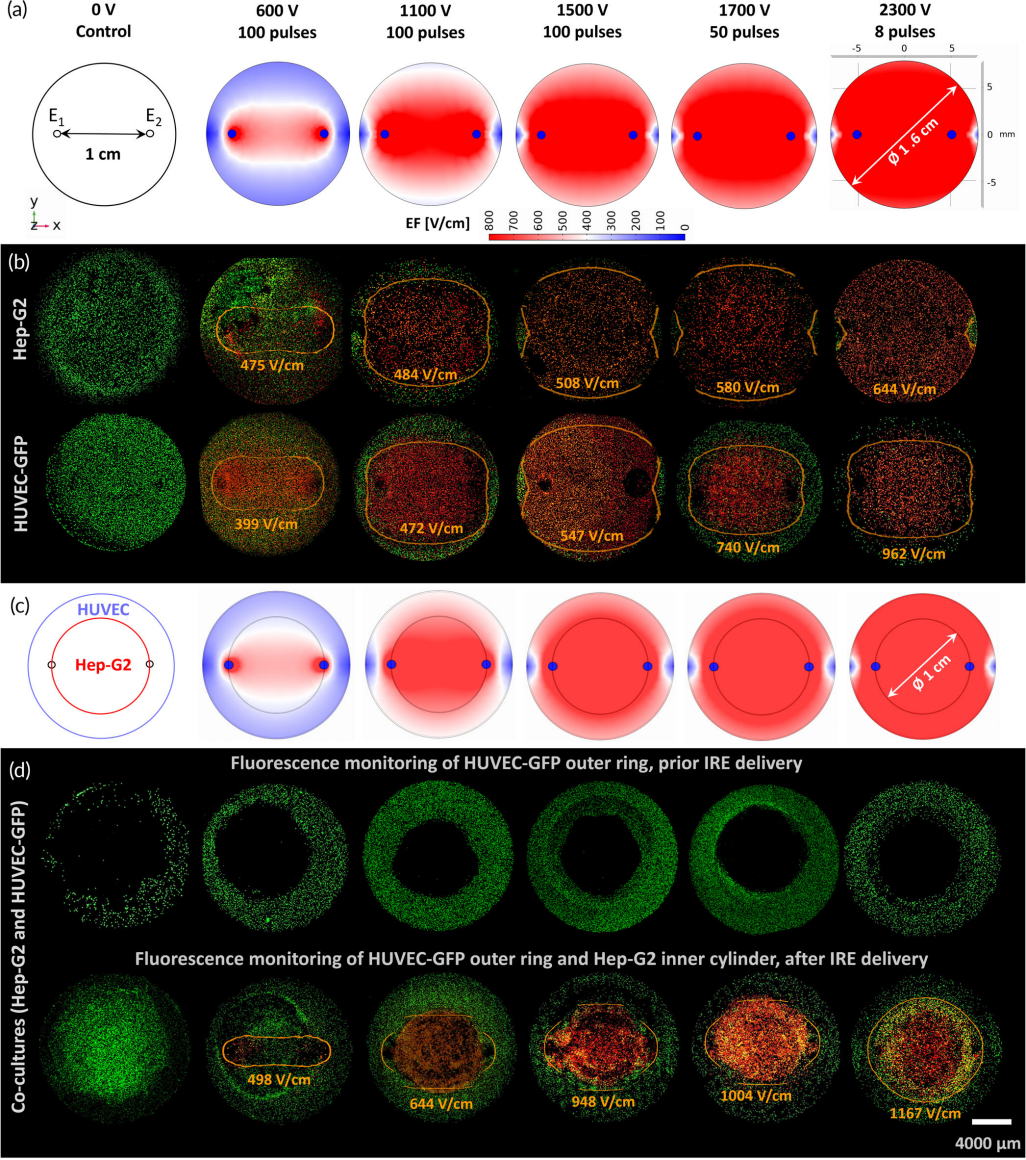
Computational modeling of IRE versus confocal imaging of small cylindrical hydrogels (two‐needle case, 24‐well plate). Geometry and IRE protocols used in (a) monocellular and (c) co‐cultured computational models. Live/dead assay outcomes for (b) Hep‐G2, HUVEC‐GFP, and (d) co‐cultured hydrogels along with the electric field (EF) iso‐surface calculated for the ablation region.

The EF distribution provoked in 24‐well monocultured and co‐cultured hydrogels by IRE appear in Figure 3a,c. A sharp difference on the EF patterns between monocultured and co‐cultured hydrogels may be observed. This is explained by the different geometrical composition of the scaffolds and the electrical conductivity of the regions comprising the hydrogels. The ablation extents reported in Figure 2a,b are graphically depicted by confocal imaging in Figure 3b,d. Therein, the cell damage after IRE appear stained in red, and the viable cells in green. The absence of electric stimulation (0 V), considered as the control condition, exhibits the preservation of cell viability in the constructs. Furthermore, the EF iso‐contours fitting the ablation area overlap the confocal images. They denote that the lower the number of pulses, the stronger the EF is required to cause cell death. The final EF threshold was 544 ± 61 V/cm for Hep‐G2 cells.

As observed in Figures 3b,c, 600 V‐100 pulses exhibited a reduced ablation extent in Hep‐G2 (26% of the total area), HUVEC‐GFP (33%), and co‐cultured (20%) hydrogels, in accordance with the corresponding computational EF. As predicted in silico, 1100 V‐100 pulses increased the ablation zone in the three types of scaffolds (Hep‐G2: 72%, HUVEC‐GFP: 67%, co‐cultures: 39%). Moreover, 1500 and 1700 V applied in different trains of pulses in Hep‐G2 constructs elicited different effects. In terms of ablation and ΔT, the difference between 1500 V‐100 pulses (92%, 18.5°C) and 1500 V‐50 pulses (88%, 14.2°C) was statistically insignificant, though the final temperatures tended to increase ($T_{f\left(1500\;V-100\;p\right)}=41.6{}^{\circ}C,T_{f\left(1500\;V-50\;p\right)}=35.9{}^{\circ}C$). Ensuring a low ΔT is important since a temperature above 50°C triggers a denaturation process in the Matrigel proteins. As for 1700 V, the comparison among 8, 50, and 100 pulses, in terms of ablation (88%, 93%, and 85%), showed that only 50 pulses are statistically significant. Conversely, ΔT (4.9, 14.9, and 22.9°C) was significantly different among all pulse trains related to the final temperatures ($T_{f\left(1700\;V-8\;p\right)}=28.3{}^{\circ}C,T_{f\left(1700\;V-50\;p\right)}=36.7{}^{\circ}C,T_{f\left(1700\;V-100\;p\right)}=46.2{}^{\circ}C$). Therefore, three IRE protocols provoked an efficient ablation in 24‐well Hep‐G2 hydrogels, while preserving their thermal stability: 2300 V‐8 pulses, exhibited the maximal ablation (97%) in the shortest time (8 s), with the lowest ΔT; 1700 V‐50 pulses were statistically comparable to 2300 V‐8 pulses in terms of ablation; and 1500 V‐100 pulses were comparable to 1700 V‐50 pulses. Hence, the ablation extent in Hep‐G2 hydrogels is strongly dependent of the pulse amplitude.

Unlike Hep‐G2 cells, endothelial cells were overall more resistant to IRE since HUVEC‐GFP‐seeded hydrogels exhibited smaller ablation areas when undergoing the same treatment conditions applied to Hep‐G2 scaffolds (Figures 2b and 3b). Contrary to Hep‐G2, the number of pulses in HUVEC‐GFP scaffolds plays a more impactful role in the ablation extent than the pulse amplitude. Treatments comprising 100 pulses generated a comparable ablation extent in both Hep‐G2 and HUVEC‐GFP scaffolds, regardless of the pulse amplitude. However, the treatment exhibiting the largest ablation in HUVEC‐GFP hydrogels was 1500 V‐100 pulses (89%) over 1700 V‐50 pulses (64%) and 2300 V‐8 pulses (68%).

Co‐cultured hydrogels in Figure 3c were scanned before and after IRE by confocal microscopy. No staining was needed before IRE delivery for monitoring the naturally‐fluorescent HUVEC‐GFP cells within the external ring, but they were stained after IRE to evaluate cell viability in both cell types. Figures 2b and 3d denote a remarkable decrease in the ablation extent when hydrogels are seeded with HUVEC‐GFP and Hep‐G2. Endothelial cells showed to be mostly non‐responsive to IRE, whereas the Hep‐G2 region is entirely ablated for a voltage above 1100 V. The most efficient treatments in co‐cultured hydrogels, being statistically comparable, were 2300 V‐8 pulses and 1700 V‐50 pulses, generating the largest ablation area, 52% and 43%, respectively.

### 2.3 Effects of IRE on 12‐well hydrogels treated with three needles

**FIGURE 4:**
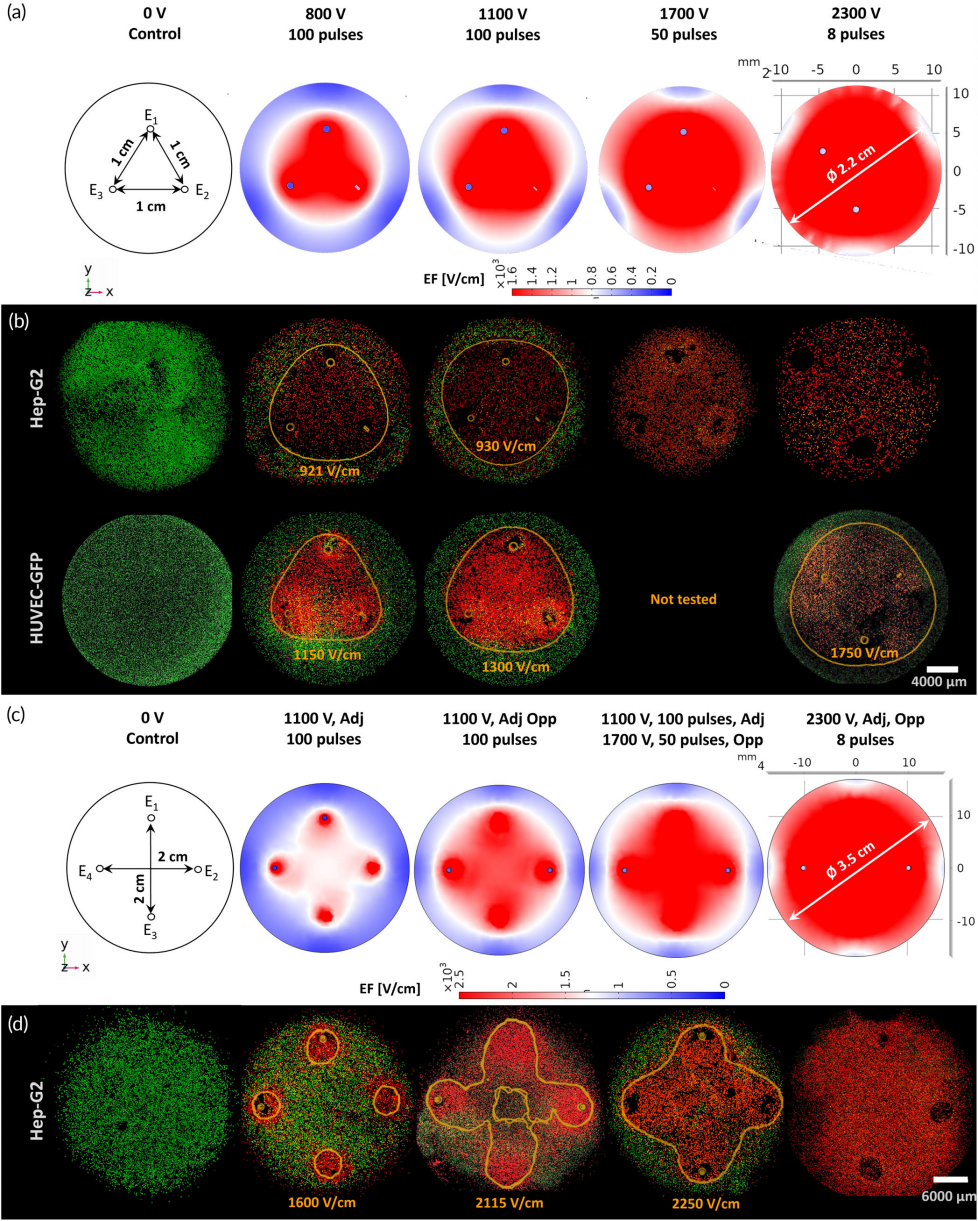
Computational modeling of IRE versus confocal imaging of medium (three‐needle case, 12‐well plate) and large (four‐needle case, 6‐well plate) monocellular cylindrical hydrogels: Geometry and IRE protocols used in (a) medium and (c) large computational models. Live/dead assay outcomes for (b) Hep‐G2 and HUVEC‐GFP medium scaffolds, and for (d) Hep‐G2 large scaffolds along with the electric field iso‐surface calculated for the ablation region.

IRE protocols were delivered thrice, that is, the same pulse amplitude between every needle pair (Figures 2c and 4a). Overall, the ablation area increased proportionally to the pulse amplitude (Figure 4b); 800 V‐100 pulses induced the smallest ablation area in both, Hep‐G2 (38%) and HUVEC‐GFP (43%) hydrogels; 1100 V‐100 pulses slightly enlarged the ablation extent in both cell‐type scaffolds to 56% and 55%, respectively. In terms of ablation and ΔT, these two protocols were statistically comparable; 1700 V‐50 pulses were delivered to Hep‐G2 hydrogels causing 64% ablation, which was statistically comparable to 1100 V‐100 pulses. However, 1700 V‐50 pulses were discarded in HUVEC‐GFP hydrogels since it induced the highest ΔT (25°C), reaching 47°C, close to the temperature threshold for protein denaturation; 2300 V‐8 pulses caused the maximal ablation in both, Hep‐G2 (86%) and HUVEC‐GFP (75%) constructs. However, this latter protocol showed a statistically significant difference between cell types. Therefore, as found in 24‐well hydrogels, endothelial cells showed to be more sensitive to the number of pulses than Hep‐G2 cells.

### 2.4 Effects of IRE on six‐well hydrogels treated with four needles

Four treatments were tested in six‐well hydrogels (Figure 4c) combining pulse amplitudes considered as effective in Sections 2.2 and 2.3. As depicted in Figure 4d, 1100 V‐100 pulses between adjacent pairs provoked a negligible ablation (17%); the same protocol delivered to the six electrode pairs, considerably enlarged the necrosis area to 49%; 1100 V‐100 pulses and 1700 V‐50 pulses, between adjacent and opposite electrodes respectively, increased it up to 65%; 2300 V‐8 pulses, between the six electrode pairs, produced the maximal ablation area (89%). The treatments maintained the temperature of hydrogels below the denaturation threshold, that is, $T_{f\left(1100V-100p,\mathrm{Adj}\right)}=25.60{}^{\circ}C$, $T_{f\left(1100V-100p,6\;\text{pairs}\right)}=27.4{}^{\circ}C,T_{f\left(1100V-100p,\mathrm{Adj},1700V-50p,\mathrm{Opp}\right)}=30.50{}^{\circ}C$, and $T_{f\left(2300V-8p,6\;\text{pairs}\right)}=26.50{}^{\circ}C$, with an average ΔT = 5°C. Figure 2e exhibits the dependence of the ablation area in large Hep‐G2 hydrogels, on the number of active pairs and the pulse amplitude.

### 2.5 Effects of IRE on tumor‐shaped hydrogels

**FIGURE 5:**
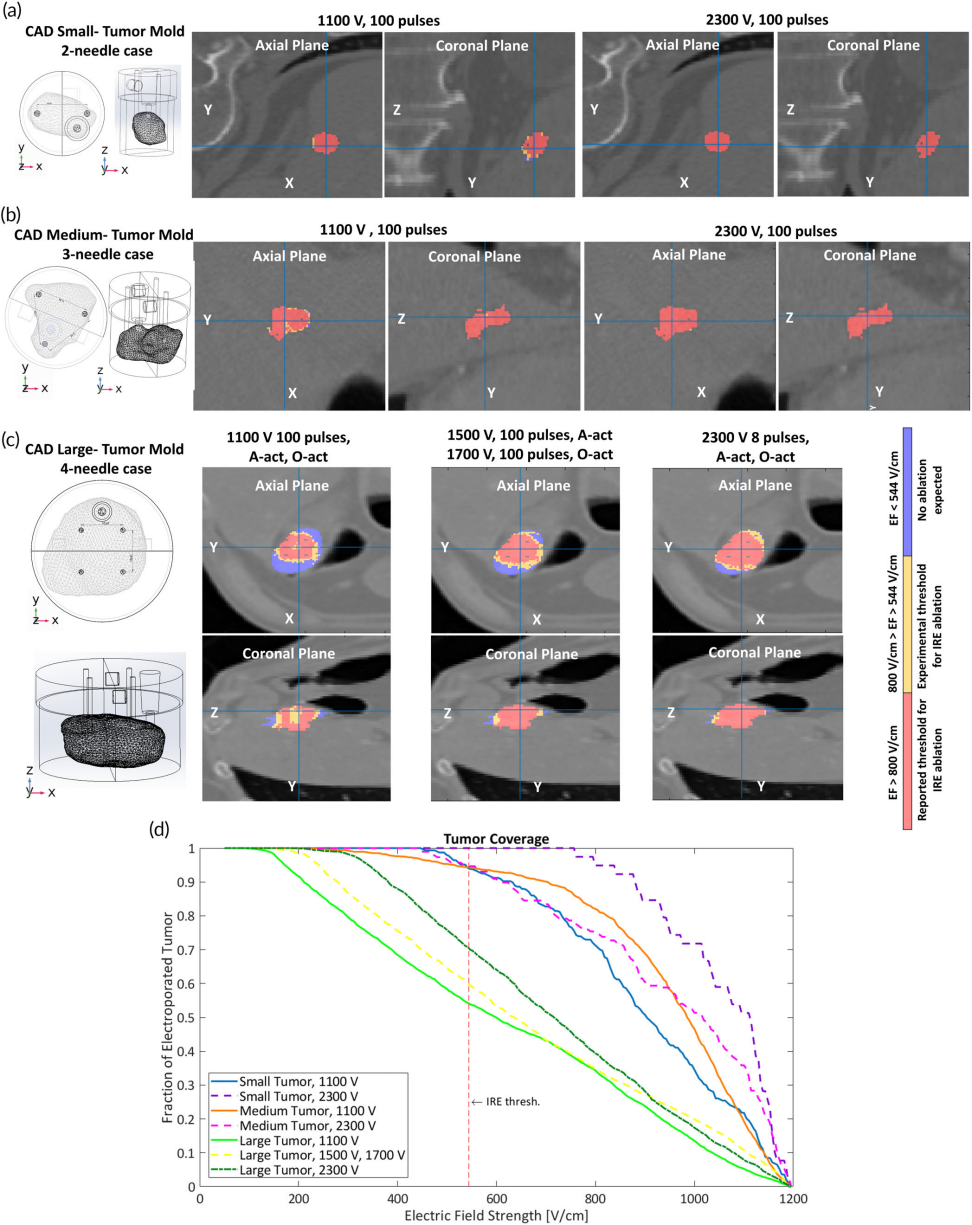
Computational planning of IRE in realistic tumors. IRE color map overlapped on CT images of a (a) small, (b) medium, and (c) large tumor. EF strength below the experimental threshold (544 V/cm) is considered as untreated (blue). IRE coverage is shown in yellow and orange for experimental (EF > 544 V/cm) and reported (EF > 800 V/cm) thresholds, respectively. (d) Normalized coverage of the three tumors simulated for the IRE treatments.

Figure 5 depicts the IRE calculated for realistic tumor models in silico. The EF distribution appears as a color map overlaid on CT images of a small, medium, and large tumor (Figure 5a–c). Colors in axial and coronal planes denote: regions exposed to an EF below the experimental IRE threshold (${\mathrm{EF}}_{\text{ExpIRE}}=$544 V/cm) marked in blue, where no ablation is expected; the EF above ${\mathrm{EF}}_{\text{ExpIRE}}$, but below the IRE‐EF threshold (${\mathrm{EF}}_{\text{RepIRE}}=$800 V/cm) reported in‐vivo, in animal models of healthy liver, appears in yellow; and the EF above ${\mathrm{EF}}_{\text{RepIRE}}$ in orange. Ablation between 94% and 100% ($\mathrm{EF}>{\mathrm{EF}}_{\text{ExpIRE}}$) in small tumors, was predicted for 1100 V‐100 pulses, and 2300 V‐8 pulses, respectively. Similarly, 94% and 95% ablation of medium tumors, treated with three needles, were expected for 1100 V‐100 pulses, and 2300 V‐8 pulses, delivered thrice. The ablation computed for large tumor hydrogels (Figure 5c) was: 54% for 1100 V‐100 pulses (Adj. activation); 60% for 1500 V‐100 pulses (Adj. activation) plus 1700 V‐100 pulses (Opp. activation); and 70% for 2300 V‐8 pulses (Adj, Opp activation). The normalized fraction of hydrogel tumors undergoing these treatments is shown in Figure 5d, where 1 represents a complete coverage (100%) of a tumor. The most ablative treatment in tumor‐shaped scaffolds was 2300 V‐8 pulses, coinciding with Hep‐G2 cylindrical hydrogels.

**FIGURE 6:**
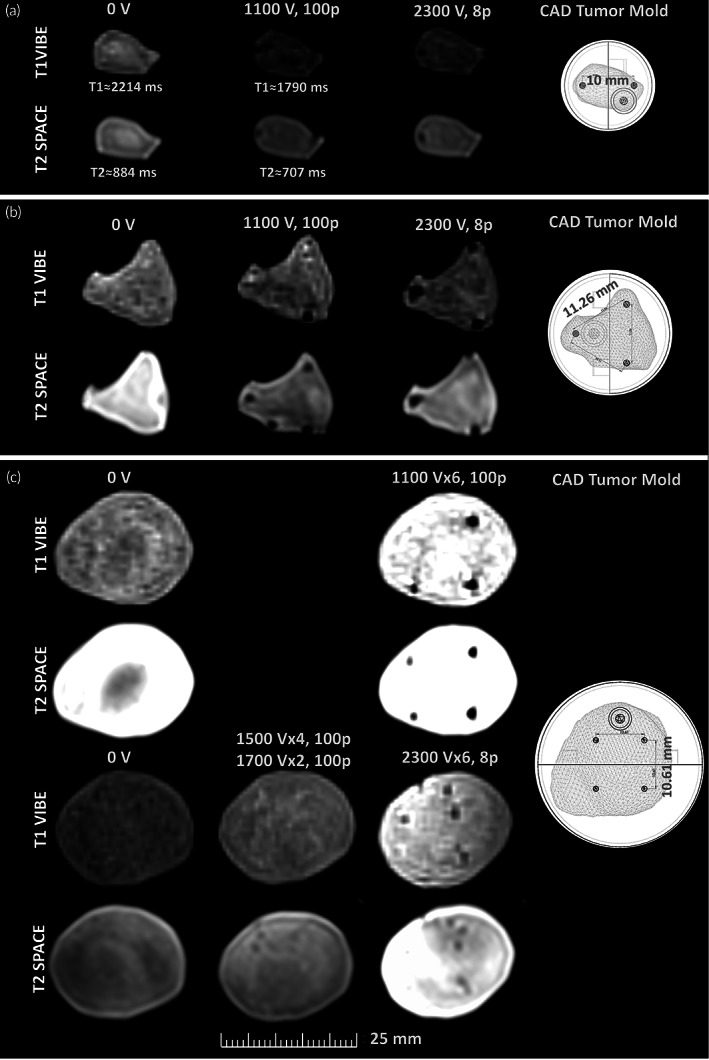
T1‐weighted VIBE and T2‐weighted SPACE MR images of (a) small, (b) medium, and (c) large tumor‐shaped hydrogels, 24 h after IRE. Images acquired along parallel and perpendicular orientation to the electrode insertion. The distance between the needle–electrodes appears in the CAD molds. Untreated tumors were utilized as control conditions. Small and medium treated tumors were hypointense in T1‐weighted VIBE images and hyperintense in T2‐weighted SPACE images, 24 h after IRE. Oppositely, inverting evolution of the SI of large tumors from hyperintense, within the first 24 h after IRE is visualized.

Experimentally, the effects of IRE on these constructs were visualized by T1‐weighted (VIBE) and T2‐weighted (SPACE) MRI sequences (Figure 6). Untreated tumors were utilized as control conditions. The insertion sites of electrodes appear as signal voids in both, confocal and magnetic resonance (MR) images, which become larger as the EF strength increases, due to loss of hydrogel caused by coagulative necrosis around the electrodes. Signal intensity (SI) in MR images of tumors in Figure 6 varies in all tumor sizes depending on the treatment. T2‐weighted (SPACE) exhibit a higher SI than T1‐weighted (VIBE) images. This is attributed to the fluid‐based composition of the hydrogels. Furthermore, a reduction in relaxation times was estimated, through a tabletop MRI scanner, in small tumors undergoing IRE (T1 = 1790 ms, T2 = 707 ms), compared to untreated tumors (T1 = 2214 ms, T2 = 884 ms), which corroborates the loss of matter in treated samples (see Table SI).

## 3 DISCUSSION

Dense collagen I and hyaluronic acid hydrogels, seeded with pancreatic cancer, and glioblastoma (GBM) cells, have been reported to evaluate IRE efficacy in vitro, in 10‐mm length and 1‐mm height, circular and squared scaffolds, treated with two‐needle electrodes.^10^, ^17^, ^18^, ^19^ Herein, larger hydrogels containing Hep‐G2 cells demonstrated to be an effective means to mimic the HCC microenvironment for evaluating the accuracy of pre‐planned IRE with multiple electrodes. Moreover, tumoral and endothelial cells in the hydrogels provide cellular components promoting an angiogenic environment, which is one the most important hallmarks of cancer.^20^, ^21^, ^22^ To circumvent the complexity of fully replicating the tumoral microenvironment, HUVEC cells concentrically embedded a cylinder of Hep‐G2 cells, representing the presence of endothelial cells which showed a migratory phenotype in fluorescence microscopy. The disposition of cells in co‐cultures aimed at mimicking the increased peripheral vasculature exhibited in a tumor, compared to its hypoxic center lacking blood vessels. Not only were HUVEC cells used as non‐malignant phenotype, but as an angiogenic promoter conferring invasive features. The higher demand of voltage to eradicate endothelial cells, compared to liver‐tumor cells, supports the findings reported for non‐transformed BEAS‐2B cells.^23^ The poor response of endothelial cells to IRE, particularly exhibited in co‐cultured hydrogels, aligns with its reported significant impact on the EF distribution.^24^ This trend may be observed in Figures 2b and 3d, and Figure S1, for an alternative experiment in which the HUVEC‐GFP hydrogel was pipetted directly in the well center resembling a semi‐sphere. Therein, the ablation zone diminishes concentrically through the Hep‐G2 hydrogel from the bottom to the top, whereas the viable extent becomes inversely larger through the HUVEC‐GFP hydrogel.

The EF threshold for IRE in liver tissue has been reported in non‐cancerous animal models of rats and porcine as: 637 ± 43 V/cm for exposures to eight, 100 μs pulses delivered at 1 s^9^; 300–500 V/cm to a single 20 ms pulse of 1000 V/cm^8^; and 423 V/cm at 1 and 4 s.^11^ In this regard, our resultant EF threshold in cylindrical hydrogels (544 ± 61 V/cm) for Hep‐G2 cells is comparable to the literature if we consider that non‐cancerous tissues demand a higher EF, as demonstrated for HUVEC‐GFP hydrogels.

Opposite to previous investigation on single focal HCC,^7^, ^13^ the IRE protocols herein were computationally established before delivery. The tumor‐shaped hydrogels behaved as useful means to monitor post‐IRE ablation by MRI. Small and medium treated tumors were hypointense in T1‐weighted (VIBE) images and hyperintense in T2‐weighted (SPACE) images, 24 h after IRE, as reported for tumors ranging from 0.9 to 2.2 cc^3^.^25^, ^26^, ^27^ However, at the same timepoint, large tumors exhibited a higher SI in both, T1‐weighted (VIBE) and T2‐weighted (SPACE) sequences compared to the untreated samples, as referenced for large tumors (~8.5 cc^3^) monitored after an early (24 h) post‐IRE period.^21^ Our findings support the inverting evolution of the SI of large tumors from hyperintense, within the first 24 h after IRE, to hypointense in Zhang et al.,^25^ Figini et al.,^26^ and Barabasch et al.^27^ We attribute the SI behavior to the tumor size since the number of cells significantly vary from a small tumor (7 × 10^5^) to a large tumor (4 × 10^6^). In these latter, the local edema due to ionic leakage induced by the irreversible permeabilization of the membrane is visualized under MRI before a complete necrosis is evident, as visualized in small and medium tumors. These findings show the efficacy of IRE planning in silico, since more that 94% of tumor ablation was estimated for small and medium tumors. An incomplete ablation predicted for large tumors was experimentally corroborated, and is explained by the constrained positioning of the needles inside the rigid molds. The agreement of our outcomes in vitro and in silico support recent evidence of the negligible effect of liver microstructure on the planning of the EF distribution for IRE^18^ and a correct assumption of boundary conditions in our models. In this manner, the appropriate treatment parameters may be tuned in vitro rather than in vivo, as recommended in Arena et al.^10^

Our investigation builds on exhibiting the assets of IRE as an effective minimally‐invasive ablation procedure based on a treatment planning that emphasizes a precise ablation, aiming at preventing either reversible electroporation due to the application of a lower voltage, or thermal damage caused by a higher voltage.^1^, ^4^

## 4 MATERIALS AND METHODS

3D‐cell‐cultures have been utilized as a powerful experimental tool for investigating tumor progression, and IRE in vitro^10^, ^17^, ^18^, ^19^, ^28^, ^29^, ^30^, ^31^ since they confer appropriate polarity to cells and mimic the elements in the extracellular matrix (ECM), which influence cell differentiation, proliferation, migration, and shape.^28^, ^31^, ^32^ Moreover, they incorporate the interaction between cells and between a cell and the ECM that in vivo tumors comprise.^19^, ^28^, ^30^, ^31^

Hydrogels are a subtype of 3D‐cell‐culture composed of polymer networks seeded with cells that solidify after gelation of a liquid precursor solution. They are mechanically similar to soft tissues, support cell adhesion, and their optical transparency enables in situ cell microscopy imaging.^32^, ^33^

### 4.1 In vitro setup

#### 4.1.1 Cell cultures

Hep‐G2 cells (derived from human HCC) were obtained from ECACC 85011430 and cultured in Dulbecco's Minimum Essential Medium (DMEM; Sigma‐Aldrich D6546, Germany) supplemented with 10% fetal bovine serum (FBS; Sigma‐Aldrich F9665, Germany), 2 mM L‐glutamine (Sigma‐Aldrich G7513, Germany), and 100 U/mL penicillin/100 μg/mL streptomycin (Sigma‐Aldrich P4333, Germany). HUVEC cells, derived from primary human umbilical vascular endothelial cells (Lonza, Belgium), were provided by the Department of Biomedical Engineering, (University of Twente, Enschede, The Netherlands) and were genetically modified to contain green fluorescent protein (GFP), which acts as a marker naturally visualized under microscopy. They were cultured in Endothelial Cell Growth medium v2 with a supplement kit (cat no: C‐22011, Promo Cell, Merck). HepG2 and HUVEC‐GFP cells were cultured at 37°C in 5% CO_2_ in a humified incubator (Heracell 150i). Matrigel (Corning, CAT‐354234, Merck) was used as the ECM for the cells within the hydrogels, since it is one of the most common ECM used in cancer research for tissue‐mimicking culture modeling.^34^, ^35^ It is extracted from the Engelbreth‐Holm‐Swarm (EHS) mouse sarcoma and comprises 60% laminin, 30% collagen IV, 8% entactin, heparan sulfate proteoglycans, and growth factors,^32^ and its concentration varies from batch to batch (8.5 mg/mL to 9.5 mg/mL).

#### 4.1.2 Preparation of hydrogel solutions

**FIGURE 7:**
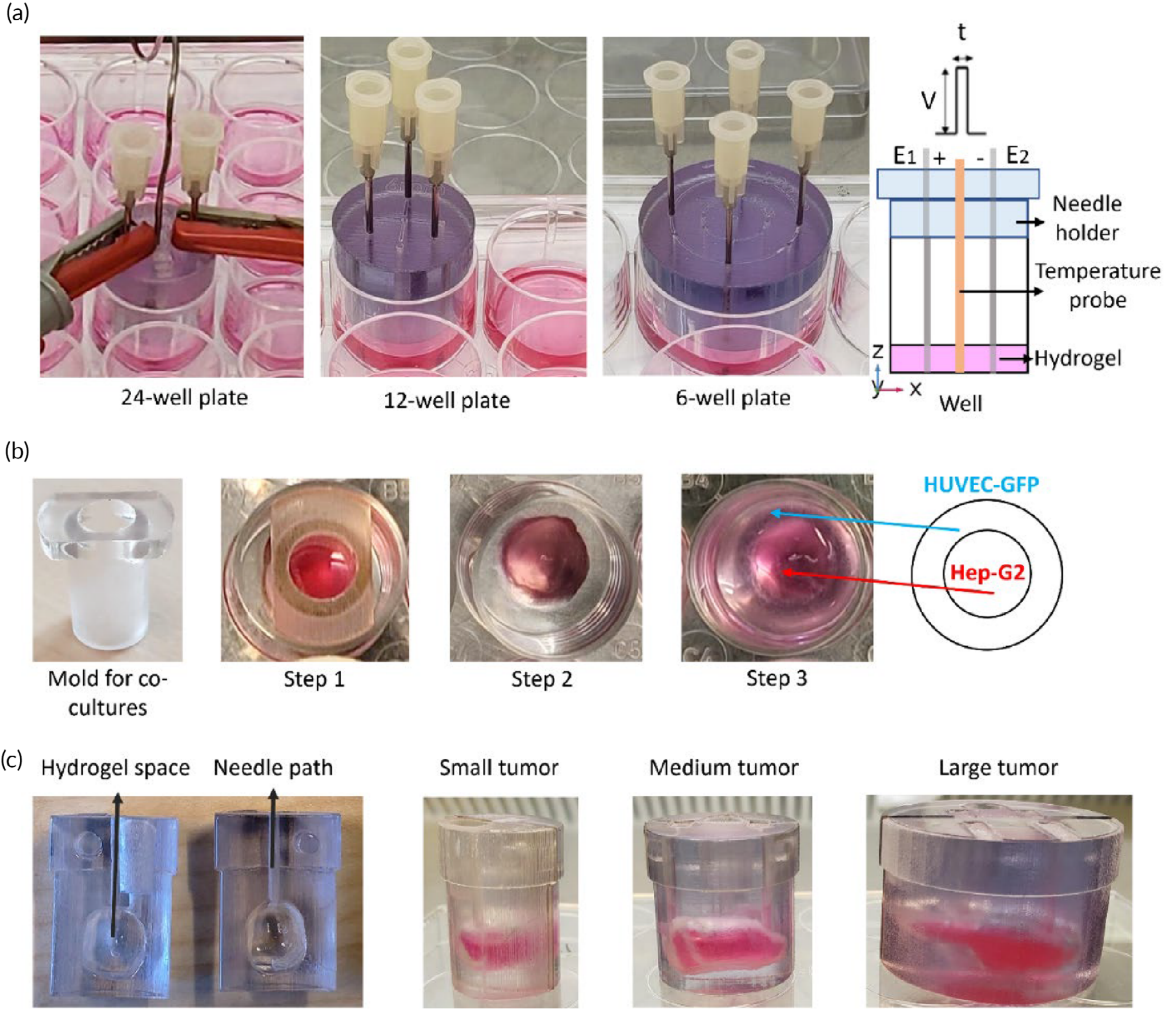
Synthesis of (a) cylindrical monocellular hydrogels, (b) co‐cultured hydrogels (Hep‐G2 plus HUVEC‐GFP), and (c) tumor‐shaped Hep‐G2‐seeded hydrogels.

The matrix of hydrogels consisted of a mixture of stock Matrigel, cells, and their corresponding culture medium in accordance with the protocol described by Corning.^34^, ^35^ The cells were trypsinized, centrifuged, and resuspended in chill medium to reach the final cell density. The cell suspension was mixed delicately with the calculated volume of stock Matrigel, for the final concentration to be 5, 6, and 7 mg/mL for monocellular (Figure 7a), co‐cultured (Figure 7b), and tumor‐shaped hydrogels (Figure 7c), respectively. The matrices were prepared on ice to prevent polymerization of the Matrigel and poured into pre‐cooled well plates by reverse pipetting to minimize the formation of air bubbles. Afterwards, the matrices were kept at 37°C in 5% CO_2_, for gelation. The hydrogels were treated with IRE, 24 h after polymerization.

#### 4.1.3 Synthesis of monocellular hydrogels

Hep‐G2 and HUVEC‐GFP cells were seeded independently in monocellular hydrogels to compare the response of liver cancer cells and endothelial cells to IRE. These hydrogels were fabricated in three different volumes (0.4 , 1.0, and 2.2 cm^3^) and poured into pre‐cooled 24‐, 12‐, and 6‐well plates to resemble small, medium, and large cylindrical tumors, as shown in Figure 7a, with a corresponding cell density of 4.8 × 10^5^ cells/well, 1.2 × 10^6^ cells/well and 2.6 × 10^6^ cells/well (Table SII).

#### 4.1.4 Synthesis of co‐cultured hydrogels

Co‐cultured hydrogels were synthetized within 24‐well plates, in a three‐step process (see Figure 7b, Table SII): (1) 0.2 cm^3^ of hydrogel matrix, seeded with 2.5 × 10^5^ Hep‐G2 cells, were poured inside a mold printed in Veroclear (polymethyl methacrylate, PMMA) placed in the center of the well and kept in a humified incubator during 30 min until its polymerization; (2) the inner hydrogel was smoothly detached from the mold; and (3) finally, 0.4 cm^3^ of hydrogel matrix seeded with 2.5 × 10^5^ HUVEC‐GFP cells were poured into the free surface of the well surrounding the inner Hep‐G2 core for further gelation. The morphology of cells in both, culturing flasks and within the Matrigel, under light microscopy, is shown in Figure S2.

#### 4.1.5 Synthesis of hydrogel‐based tumors

Three molds were printed in MRI‐compatible, autoclavable Veroclear to contain tumor‐shaped hydrogels (Figure 7c). These molds fitted 24‐, 12‐, and 6‐well plates containing 0.6, 1.1, and 3.4 cm^3^ of hydrogels with a cell density of 7 × 10^5^, 1 × 10^6^, and 4 × 10^6^ Hep‐G2 cells/mold, respectively (see Table SII). The tumor‐shaped hydrogels correspond to 3D reconstructions of tumor segmentations of CT images of three patients with liver cancer.

### 4.2 IRE with multiple needles

Square electric pulses were delivered with a BTX™ Gemini X2 electroporator. Blunt‐tip G19 stainless steel needles, 0.91 mm outer diameter (OD), were utilized as electrodes for pulse delivery. Needle holders were designed in SolidWorks and printed in Veroclear (PMMA) for insertion and immobilization of the electrodes during the treatment (Figure 7a). The IRE treatments were delivered with two, three, and four needles, denoted as E_1_, E_2_, E_3_, and E_4_, in small, medium, and large tumors, respectively. The IRE treatments per sample were replicated thrice independently (summarized in Figure 2 and Table SIII). The temperature was monitored with a digital thermometer in the cylindrical hydrogels (Figure 7a), and with a FLIR ONE thermal‐imaging camera in the hydrogel tumors.

### 4.3 Ablation assessment

The ablation was visualized in two manners: by confocal microscopy in cylindrical hydrogels, with a Nikon A1 confocal laser microscope system (Bioimaging Centre, University of Twente); and by MRI in tumor‐shaped hydrogels at 1.5 T (Magnetom Aera, Siemens Healthineers, Erlangen, Germany), and 0.5 T (Tabletop scanner, MagSpec, Pure Devices, Rimpar, Germany) field strength.

#### 4.3.1 Live/dead assay for confocal imaging

Fluorescent dyes, Calcein AM (1 μg/μL; Invitrogen C3100MP, Fisher Scientific, Germany) and propidium iodide (PI; 3.75 μg; Molecular Probes P3566, Fisher Scientific, Germany), were utilized for monitoring living cells in green and dead cells in red, respectively. Twenty‐four‐well, 12‐well, and 6‐well hydrogels were stained and incubated during 40, 90, and 180 min, respectively. Three 24‐well hydrogels were treated and followed‐up 3, 24, and 48 h after IRE, to determine the suitable scanning timepoint. No change in the ablation zone was found. Therefore, all subsequent samples were imaged 48 h after IRE. 3D‐views of the treated hydrogels were obtained by scanning z‐stacks, that is, slices of the 24, 12, and 6 wells along a 2 mm thickness. The ablation surface was measured with ImageJ along the z‐stacks to calculate the corresponding unviable volume in the hydrogel, and hence the coverage with IRE.

#### 4.3.2 MRI sequences

The MR images were acquired along parallel and perpendicular orientation to the electrode insertion, 24 h after treatment with IRE. The hydrogels were not scanned prior to treatment to preserve their sterility. T1‐weighted Volumetric Interpolated Breath‐hold Examination (VIBE), and T2‐weighted three‐dimensional isotropic turbo spin‐echo, Sampling Perfection with Application optimized Contrast using different flip angle Evolution (3D‐SPACE) sequences were performed for a thin continuous acquisition based on the size of the samples. Estimation of T1 and T2 relaxation times was carried out for small tumors using inversion recovery and multi echo spin echo sequences on a 0.5 T tabletop MRI scanner. Since this scanner is designed for small biopsied tissues (bore diameter = 1.5 cm), medium and large tumors were not considered for this analysis. The setup for scanning small hydrogels tumors with the tabletop scanner is provided in Figure S3.

### 4.4 Statistical analysis

Experimental results in Figure 2 are reported as mean ± standard deviation from the replications per sample. Two‐way ANOVA was performed to determine the significant difference in means (*p < 0.05; **p < 0.01; ***p < 0.001). Alongside, a Tukey's multiple comparisons test was used to determine the correlation between the pulse amplitude, the number of pulses, and the ablated region in the different hydrogel setups. Statistical analysis was performed using GraphPad Prism (version 10.0.0 for Windows, GraphPad Software, Boston, MA).

### 4.5 Computational modeling

#### 4.5.1 Determination of trial IRE protocols in small cylindrical hydrogels

The baseline pulse amplitudes for IRE were determined for 24‐well cylindrical hydrogels by the finite element method. The needle‐electrodes were modeled by 3 mm‐height stainless‐steel cylinders, and the hydrogel as the largest cylinder (geometry in Figure 3a). The electrical conductivity of the hydrogel was programmed as an EF dependent variable to emulate its increasing behavior (σ_liv0_ = 0.4 S/m, σ_livf_ = 1.6 S/m) due to electroporation.^36^ Electric potential and ground were set as boundary conditions to every pair of electrodes, to solve the EF distribution by the Laplace equation (Equation 1) for a variety of electric potentials through a parametric study.

(1) $$\nabla ∙\left(\sigma \nabla \varphi \right)=0$$

#### 4.5.2 EF distribution in monocultured hydrogels

The resistance measured with the BTX during experimental IRE was used to calculate the conductivity in Hep‐G2 (small: 0.022–0.032 S/m, medium: 0.019–0.033 S/m, large: 0.005–0.007 S/m) and HUVEC (0.033–0.047 S/m) hydrogels. These values were reentered in the model to fit the EF distribution to the experimental setup. A surface integration was performed in the 24‐well hydrogel model to determine the ablation between 200 V/cm and 1200 V/cm, in 1 V/cm steps. The EF was solved in 12‐well and 6‐well hydrogels by sequentially activating needle pairs, and superimposing the cumulative EF generated by each active pair (Figure 4a,c). The corresponding surface integration was evaluated for every active pair of electrodes for a cumulative EF between 200 and 1600 V/cm (medium hydrogels), and 200 and 2500 V/cm (large hydrogels), in 20 V/cm steps. The results were compared with the experimental ablation area, measured with ImageJ on the confocal images, to determine the corresponding EF magnitude. The outcoming iso‐surface (orange traces on Figures 3 and 4b,d) was overlapped on the confocal images to visualize their fitting to the red ablation region.

#### 4.5.3 EF distribution in co‐cultured hydrogels

The model for this set‐up considered a cylinder (Ø = 1 cm), representing the Hep‐G2 hydrogel, embedded in an outer cylinder (Ø = 1.56 cm) simulating the HUVEC disk (Figure 3c). Conductivities for HCC and umbilical cord (1.26 S/m, 1.46 S/m) were assigned to the corresponding domains in the model.^36^, ^37^ The EF iso‐surface for the experimental ablation region was determined as described in Section 4.5.2.

#### 4.5.4 IRE planning in tumor‐shaped hydrogels

Three liver tumors were segmented from CT images and processed with Comsol Multiphysics plus LiveLink for Matlab to generate their realistic to solve the EF distribution caused by IRE. The dielectric properties and boundary conditions, described in Section 4.5.1, were conferred to the model domains. A normal‐to‐axial‐plane positioning of parallel needles was simulated in the tumor models, consistently with the needle insertion during experimentation. The activation of electrodes followed the description in Figure 2. The EF threshold calculated in Section 4.5.2 was taken into account to display the EF distribution in tumors as a color map overlaid on the original CT images, and calculate the volume of tumors expected to undergo experimental and theoretical IRE, based on visualization frameworks previously reported.^36^, ^38^

## 5 CONCLUSION

The hydrogels herein recapitulate crucial elements in the HCC microenvironment. They denote advantages over other in vitro experimental setups utilizing cell suspensions, 2D cell cultures, and vegetable models, such as the preservation of the morphology and physiological response of cells. Furthermore, these scaffolds lessen the ethical and economic hardships that in vivo experiments deploy, either in human and animal models. Moreover, these hydrogels may be beneficial over ex vivo tissues in which viability is tough to retain.

Optical and molecular properties of the constructs herein, eased the monitoring of cell viability and visualization of the cumulative EF resulting from the delivery of IRE with multiple needles. Along with the finite element modeling, confocal microscopy served to evaluate the ablation extent and the EF threshold for Hep‐G2 cells. In addition, the utilization of hydrogel‐based tumors demonstrated to be a useful reproducible in vitro tool to assess an immediate response of small and medium HCCs to IRE by MRI.

Particularly, the ablation of monocultured liver‐tumor cells exhibited a strong dependence on the pulse amplitude and a minor dependence on the number of pulses. Hydrogels between 190 and 390 mm^2^ may be completely ablated by 2300 V‐8 pulses, 100 s pulse width, 1 Hz, without a significant temperature increase. Oppositely, the ablation in monocultured endothelial cells showed to be dependent on both, the pulse amplitude and the number of pulses, reaching a maximal ablation of 89%. Either, an increase in the pulse amplitude for 50 pulses, or an increase in number of pulses for 2300 V should be tested further, since no IRE protocol provoked a complete ablation in endothelial hydrogels. Contrary to monocultured scaffolds, concentrical co‐culturing of Hepg‐G2 and HUVEC‐GFP cells elicited a sharp reduction in the ablation regions. Hep‐G2 core in co‐cultured scaffolds was most likely to be completely ablated. The disposition of cells in co‐cultures aimed at simulating the increased peripheral vasculature exhibited in a tumor, compared to its hypoxic center lacking blood vessels. In this regard, confocal microscopy exhibited a shielding effect of the HUVEC‐GFP/Hep‐G2 border, suggesting a preservation of endothelial cells after IRE and hypothetical angiogenesis.

Further investigation on the interplay between HUVEC‐GFP and Hep‐G2 is encouraged to verify the reported secretion of vascular endothelial growth factor (VEGF) and basic fibroblast growth factor (bFGF) by cancer cells,^39^ which promotes proliferation of HUVEC cells and hence, angiogenesis. Moreover, fibroblasts, regulatory and cytotoxic T cells, and tumor‐associated macrophages may be added to the hydrogels to refine their mimicry with in vivo tumors and to contribute to the production of growth factors. In addition, cellular‐destructive staining methods may be subsequently replaced by non‐invasive methods such as impedance measurements to characterize the cell viability, cell growth and differentiation, before and after delivery of IRE.

The agreement of in vitro and in silico findings exhibit the paramount importance of computational modeling in IRE. The methodology for constructing hydrogel‐based tumors allowed the planning of accurate ablation with IRE, while exhibiting its safety and efficacy in liver‐tumor mimics, in order to accelerate the translation and acceptance of IRE in clinical use.

## AUTHOR CONTRIBUTIONS

**Adriana Leticia Vera‐Tizatl:** Conceptualization (lead); data curation (lead); formal analysis (lead); investigation (lead); methodology (lead); project administration (equal); software (lead); validation (lead); visualization (lead); writing – original draft (lead); writing – review and editing (lead). **Regine van der Hee:** Formal analysis (supporting); methodology (supporting); validation (supporting); writing – review and editing (supporting). **Jeroen Cornelissen:** Formal analysis (supporting); resources (equal). **Claudia Elizabeth Vera‐Tizatl:** Formal analysis (supporting); methodology (supporting). **Momen Abayazid:** Funding acquisition (equal); project administration (equal); resources (equal); supervision (equal); writing – review and editing (equal). **Jurgen J. Fütterer:** Funding acquisition (lead); project administration (equal); supervision (equal); writing – review and editing (equal).

## FUNDING INFORMATION

The authors declare that this research received no specific grant from any funding agency in the public, commercial, or not‐for‐profit sectors.

## CONFLICT OF INTEREST STATEMENT

The authors declare no conflicts of interest.

### PEER REVIEW

The peer review history for this article is available at https://www.webofscience.com/api/gateway/wos/peer-review/10.1002/btm2.10607.

## Supporting information


**DATA S1:** Supporting Information.

## Data Availability

The data that support the findings of this study are available from the corresponding author upon reasonable request.
