# Supplementary material for: Liver‐tumor mimics as a potential translational framework for planning and testing irreversible electroporation with multiple electrodes
Source: Bioeng Transl Med. 2023 Nov 23;9(1):e10607. doi: 10.1002/btm2.10607 (PMC10771569; doi:10.1002/btm2.10607)
Supplement: Supplementary file 1 — DATA S1: Supporting Information. [file BTM2-9-e10607-s001.docx]

**Supporting Information**

**Table I. Relaxation times estimation by table top MRI scanning**

| **Estimated Relaxation Time**  **[ms]** | **Pulse Amplitude [V]**  * Common conditions: 100 pulses, 100 µs pulse width, 1 s inter-pulse delay | | | | | | | |
| --- | --- | --- | --- | --- | --- | --- | --- | --- |
|  | **Distance between needles=10 mm** | | | | **Distance between needles=7 mm** | | | |
|  | **0 V** | **0 V** | **900 V** | **1100 V** | **0 V** | **600 V** | **900 V** | **1100 V** |
| **T1** | 1957 | 2214 | 1824 | 1790 | 2205 | 1745 | 1723 | 1597 |
| **T2** | 873 | 884 | 730 | 707 | 892 | 751 | 725 | 677 |

**Table II. Hydrogel characteristics**

| **Size** | **Well type/**  **surface**  **[cm^2^]** | **Cylindrical Monocultures** | | **Cylindrical Co-cultures** | | | | **Tumor-shaped** | |
| --- | --- | --- | --- | --- | --- | --- | --- | --- | --- |
|  |  | **Vol. [cm^3^]** | **Cell density**  **[cells/well]** | **Vol. [cm^3^]** | | **Cell density**  **[cells/well]** | | **Vol. [cm^3^]** | **Cell density**  **[cells/well]** |
|  |  |  |  | **Hep-G2** | **HUVEC** | **Hep-G2** | **HUVEC** |  |  |
| Small | 24 (1.9) | 0.4 | 4.8×10^5^ | 0.2 | 0.4 | 2.5×10^5^ | 2.5×10^5^ | 0.6 | 8×10^5^ |
| Medium | 12 (3.8) | 1.0 | 1.2×10^6^ | -- | -- | -- | -- | 1.1 | 1.3×10^6^ |
| Large | 6 (9.5) | 2.2 | 2.6×10^6^ | -- | -- | -- | -- | 3.4 | 4×10^6^ |

**Table III. IRE treatment protocols**

| **IRE protocols with 2 needle electrodes** | | | | | |
| --- | --- | --- | --- | --- | --- |
| **Sample**  **Pulse**  **amplitude [V]** | **600** | **1100** | **1500** | **1700** | **2300** |
| Hep-G2 | 100 p | 100 p | 100 p  50 p | 100 p  50 p  8 p | 8 p |
| HUVEC | 100 p | 100 p | 100 p | 50 p | 8p |
| Co-cultures | 100 p | 100 p | 100 p | 50 p | 8p |
| Tumors | x | 100 p | x | x | 8p |
| **IRE protocols with 3 needle electrodes** | | | | | |
| **Sample**  **Pulse**  **amplitude [V]** | **600** | **800** | **1100** | **1700** | **2300** |
| Hep-G2 | xx | 100 p | 100 p | 50 p | 8 p |
| HUVEC | xx | 100 p | 100 p | x | 8 p |
| Tumors | xx | xx | 100 p | x | 8p |
| **IRE protocols with 4 needle electrodes** | | | | | |
| **Sample** | **Electrode activation** | | | | |
| Hep-G2 | 1100 V  A-act, 100 p | 1100 V  A-act, O-act, 100 p | 1100 V  A-act, 100 p  1700 V  O-act, 50 p | | 2300 V  A-act, O-act, 8p |
| HUVEC | 1100 V, A-act, 100 p  1500 V, O-act, 100 p | | | | |
| Tumors | 800 V  A-act, 100 p  1100 V  O-act, 100 p | 1500 V  A-act, 100 p  1700 V  O-act, 100 p | 2300 V  A-act, O-act, 8p | | |
| *p: number of pulses.*  *A-act: activation of adjacent needles (E1-E2, E2-E3, E1-E3, E3-E4).*  *O-act: activation of opposite needles (E1-E3, E2-E4).* | | | | | |


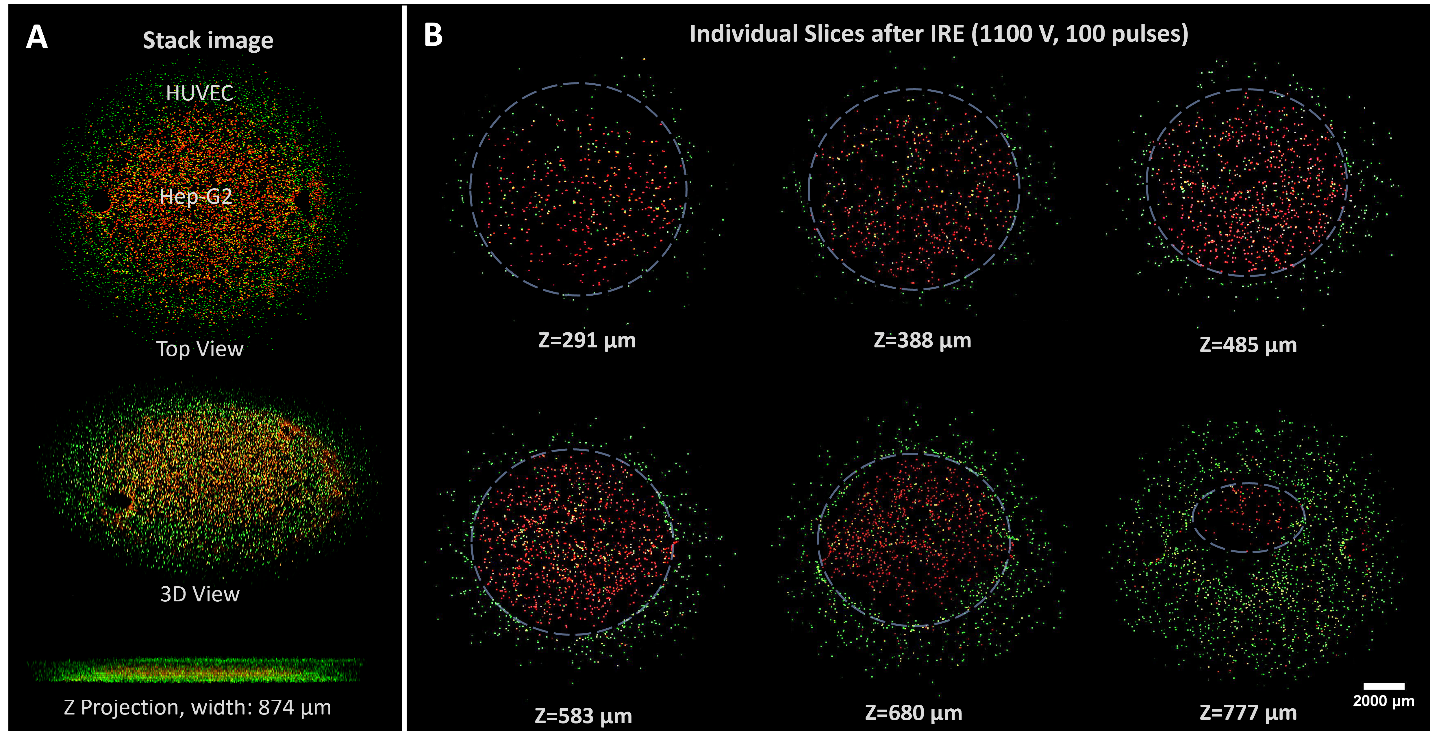


Supplementary Figure 1. Supplementary co-culture of a HUVEC-GFP hydrogel pipetted in the center of the well, resembling a semi-sphere surrounded by Hep-G2 hydrogel. A) a 3D view of this hydrogel is reconstructed from a Z-stack scanning with confocal microscopy. B) The ablation zone diminishes concentrically along the Hep-G2 hydrogel from the bottom to the top, whereas the viable extent becomes inversely larger along the HUVEC-GFP hydrogel.


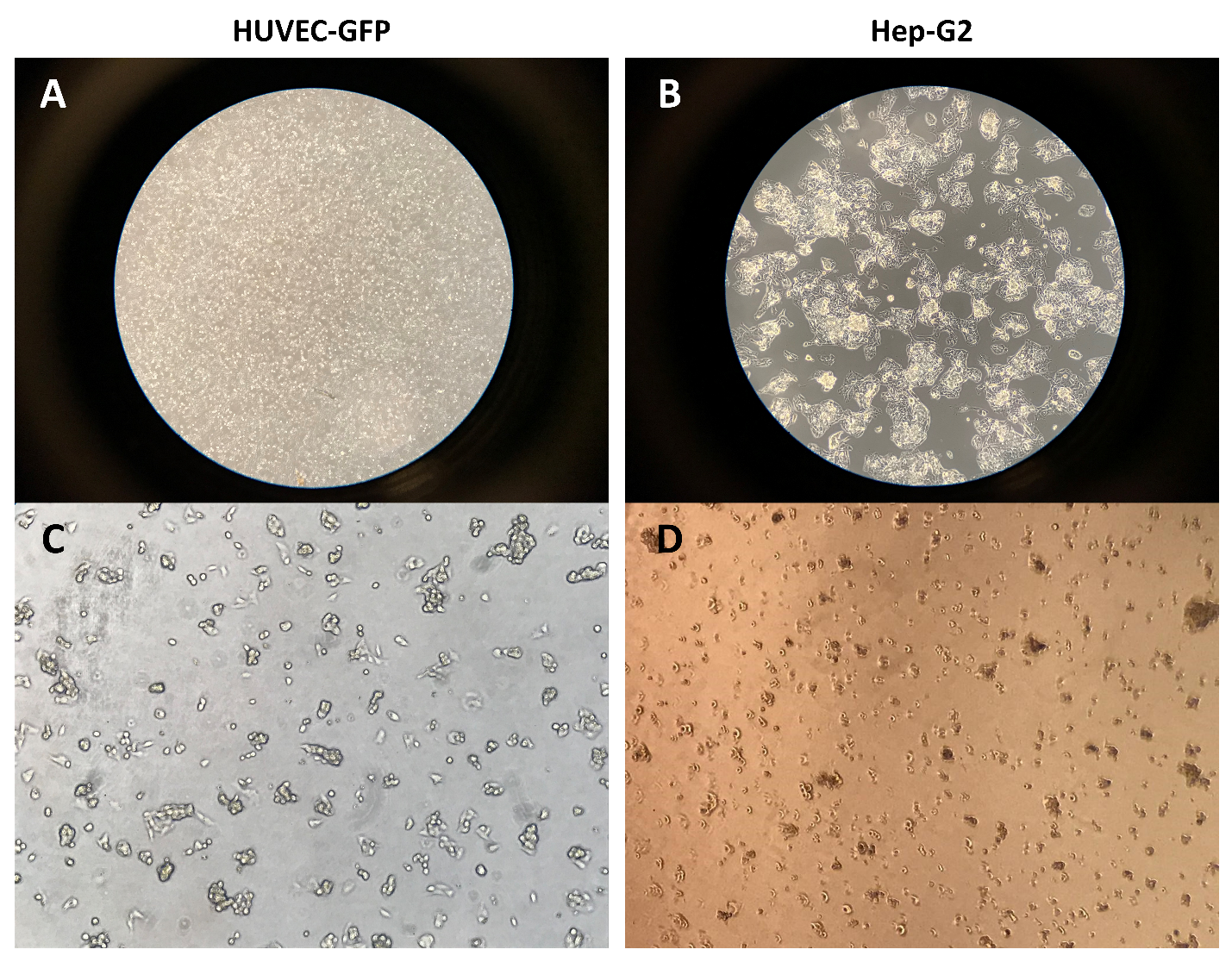


Supplementary Figure 2. Morphology of HUVEC-GFP and Hep-G2 cells. Images taken by light microscopy of fields of vision. Cell culturing of A) HUVEC-GFP (40x Magnification) and B) Hep-G2 (40x Magnification). Appearance of C) HUVEC-GFP (100x Magnification) and D) Hep-G2 (100x Magnification) in hydrogels.


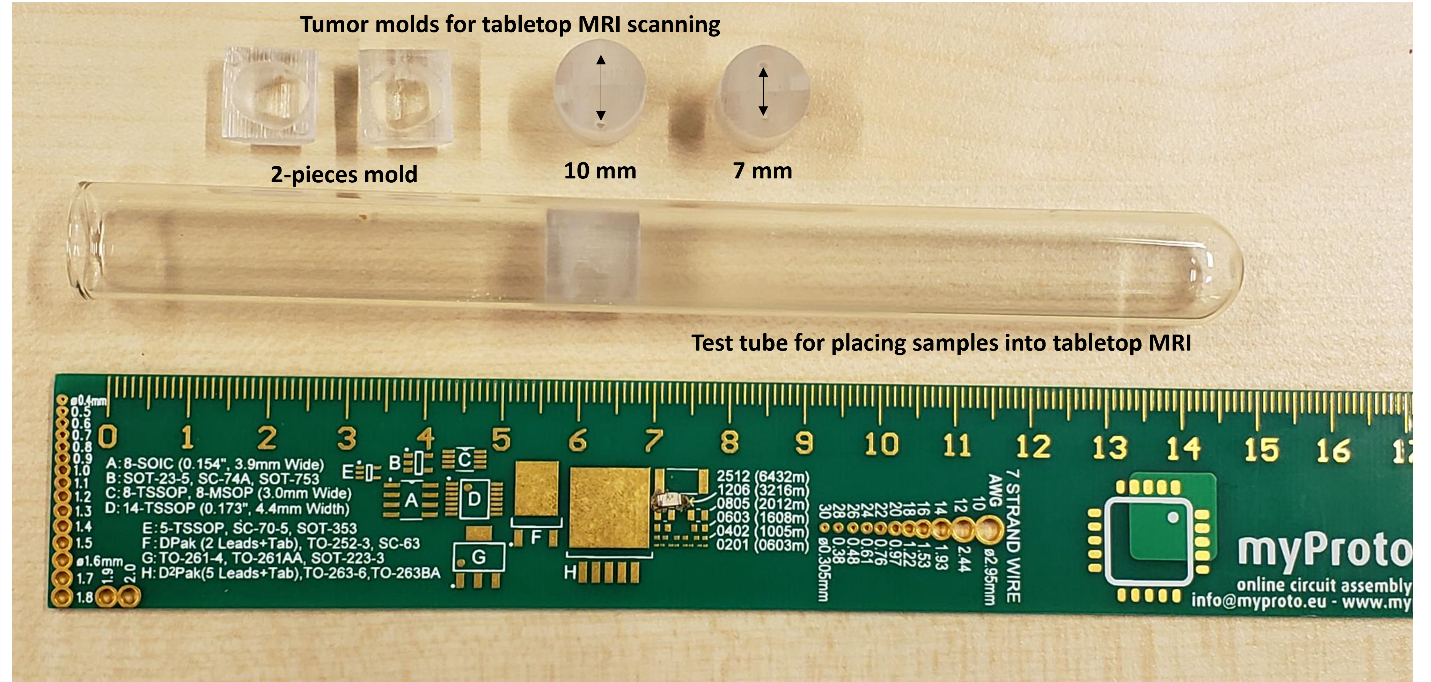


Supplementary Figure 3 Experimental set-up for scanning small hydrogel tumors with a 0.5 T tabletop MRI.
